# Money’s (not) on my mind: a qualitative study of how staff and managers understand health care’s triple Aim

**DOI:** 10.1186/s12913-017-2052-3

**Published:** 2017-01-31

**Authors:** Marie Höjriis Storkholm, Pamela Mazzocato, Mairi Savage, Carl Savage

**Affiliations:** 1 Department of Learning, Informatics, Management and Ethics, Medical Management Centre, Karolinska Institutet, Tomtebodavägen 18A, 171 77 Stockholm, Sweden; 2 Department of Obstetrics and Gynecology, Aarhus University Hospital, Aarhus, Denmark

**Keywords:** Triple aim, Mental models, Downsizing, Professions, Cost and quality, Change management, Quality improvement

## Abstract

**Background:**

The “Triple Aim” – provision of a better care experience and improved population health at a lower cost – may be theoretically sound, but paradoxical in practice as it forces together the logics of management and medicine. The aim of this study was to explore how staff and managers understand the change imperative inherent to the Triple Aim and the mental models underlying their understanding.

**Methods:**

This qualitative study builds on thirty semi-structured interviews conducted with managers, nurses, midwives, medical secretaries, and physicians at a department of Gynecology and Obstetrics in Denmark who successfully cut costs through staff and bed reductions and, from what we can ascertain, maintained care quality. Mental models were articulated from a content analysis of the interviews.

**Results:**

Staff and managers identified with the different dimensions of the Triple Aim along classic professional divides, i.e. nurses and midwives focused on patient experience, physicians on health outcomes, and manager on all three. Underlying these, we found four mental models. The understanding of change was guided by a *Professional ethos* (inner drive to improve care) and a *Socio-political discourse* (external requirement to become more efficient) mental model. The understanding of economics was guided by a *You-get-what-you-pay-for* and by a *More-bang-for-the-buck* mental model. A complex interplay could be discerned between all four, which led staff to see the Triple Aim as a dilemma between quality and economics and a threat to clinical care and quality, whereas managers saw it as a paradox that invited improvement efforts. Despite these differences, managers chose a change strategy in line with staff mental models.

**Conclusions:**

The practical challenges inherent to the Triple Aim may be symptomatic of the interactions between the different mental models that guide staff and managers’ understanding and choice of change strategies. Pursuit of quality improvement in the face of financial constraints (the essence of the Triple Aim) may be facilitated through conscious exploration of these empirically identified mental models. Managers might do well to translate the socio-political discourse into a change process that resonates with the mental models held by staff.

## Background

The Triple Aim has been described as a framework to optimize health care systems [1, 2]: To improve health care performance, organizations should aim to simultaneously improve population health, provide a better patient experience, and reduce costs [3]. The framework has found resonance and been used to guide over 100 improvement efforts in ten countries including the US and Scandinavia [2]. It is firmly rooted in an understanding of quality and cost as factors dependent on the function and design of the system, rather than solely a function of the individual skills of the people that work in that system [4].

Theoretically, this attempt to link clinical outcomes, patient experience, and cost makes sense. Deming described in his “Chain reaction” model that by investing in quality improvement (QI), costs can be reduced [5]. However, the relationship between cost and quality in health care has yet to be fully understood [6, 7].

In practice, pursuit of the Triple Aim is a challenge [2]. Only 30% of cases in a recent review have been able to improve quality and at the same time reduce costs [7]. Hospital mergers, downsizing, and building new hospitals are common strategies to increase efficiency [8, 9]. But improving quality while downsizing appears difficult [10] and can negatively affect work environment and cause burnout [11, 12]. The Triple Aim forces together goals that traditionally have appealed to two competing logics: managerialism and professionalism. Change efforts have been stymied by this conflict, even though the two logics can learn from each other [13, 14]. Some have therefore called for the development of “hybrid” managers who understand both logics [15]. All this suggests that combining money and medicine as proposed in the Triple Aim, could either be seemingly untrue (a challenging paradox), or simply “optimistically untrue” (a dilemma).

Given the magnitude of the challenge inherent to the Triple Aim, it has been argued on a conceptual level that new mental models need to be established [16]. Mental models were first defined as a “psychological representation of some domain or situation that supports understanding, reasoning, and prediction” [17, 18]. We are seldom consciously aware of them, yet they impact our behavior and often limit us to “familiar ways of thinking and acting” [19]. Through contributions from cognitive psychology, pedagogy, and organizational science, our understanding of mental models has been expanded to recognize the role of past experiences [20]. Double-loop learning occurs when we are able to articulate these mental models and become aware of their influence on how we interpret feedback and make decisions. If we do not question (and change) these “deeply held internal images of how the world works” [19], we may limit the possibility for organizations to learn about better ways to provide patient care [21]. Thus, the management of mental models, i.e. the “surfacing, testing, and improving of our internal pictures of how the world works” is a central tenant to the development of learning organizations able to adapt to societal trends, pressures, and demands [19, 22]. The Triple Aim may be theoretically sound, but it is difficult to achieve in practice. Empirical studies on the mental models underlying change efforts in the context of the Triple Aim could help managers to more deftly address the challenges. Thus, the aim of this study is to explore how staff and managers understand the change imperative inherent to the Triple Aim and the mental models underlying their understanding.

## Methods

### Study design

We employed a qualitative study design with an explorative character using semi-structured interviews.

### Study setting

This study was conducted at the Obstetrics and Gynecology (OB/GYN) department at Aarhus University Hospital (AUH), in Denmark. The Danish health care system is undergoing a major reform with the establishment and construction of sixteen new publicly funded and owned “super hospitals” [23]. As one of these, AUH now serves both as a general hospital for the 400,000 inhabitants within Aarhus County and as a highly specialized care provider for the 1.2 million inhabitants of Region Midt. It has approximately 10,000 employees, 990 beds, and an annual operating budget of €870 million.

To finance the reform, AUH was tasked to increase efficiency by 8% between 2009–2019. Management has employed mergers and downsizing-strategies related to staff and bed capacity based on demographic projections. OB/GYN was asked to reduce the budget (10%) and decrease beds (36%) and nursing staff (20%), while maintaining clinical quality and production. Thus, this case exemplifies the Triple Aim in practice, with the sub-population consisting of those served by the AUH health system.

Department management chose a lean-inspired strategy, which has been applied widely throughout health care [24]. The strategy engaged staff in interdisciplinary working groups to review 46 clinical pathways to identify potential “waste” of resources (e.g. unnecessary clinical procedures or admittances to the department) and design more efficient processes. Planning began in August 2013. By 2015, the budget had been reduced, 21 of 70 beds were closed with four more scheduled for closure by 2018, forty nurses left voluntarily (retirement or other positions), and changes were made in over 70% of the pathways. Hospital management demands were thereby successfully met. As far as we have been able to ascertain using indicators from the national OB/GYN quality registers, the department was able to maintain clinical care quality.

### Study participants

As the number of employees was large (423), we randomly selected eighteen staff from purposively chosen personnel categories. All staff was assigned a number and then randomized using an online computer program (researchrandomizer.org). Twelve managers were chosen. All had clinical backgrounds, as is the norm for clinical department managers in Denmark. To balance the greater proportion of nurses and midwives among managers, we included senior physicians and residents from each section of the department.

### Data collection

Interviews are commonly used to study mental models [20]. The first author conducted the interviews as she was both a specialist in OB/GYN and had previously worked in the study setting and therefore had extensive contextual insights. An interview guide was pilot-tested four times with physicians in OB/GYN to refine wording and ensure comprehensibility. We used open-ended questions to probe interviewees’ understanding of the purpose, objectives, content, and outcomes of the organizational changes and took into account aspects related to content, process, and context [25]. Throughout, we explored in depth juxtapositions of the Triple Aim dimensions. Interviews lasted about one hour (33–110 min) and were conducted in Danish in a quiet room at the department (except for two via Skype) between June-October 2014. All were digitally recorded.

### Data analysis

The first author transcribed the interviews *verbatim* and read through the transcripts to familiarize herself with the data. In the first analysis phase, text that mentioned any of the Triple Aim dimensions were identified as meaning units for analysis. Thereafter, a conventional inductive qualitative content analysis [26, 27] was performed in English to code, categorize, and develop themes which were organized in NVivo qualitative data analysis Software; QSR International Pty Ltd. Version 10, 2014. Codes for managers and staff were separated to identify differences. All the authors individually grouped the codes into themes in terms of staff and managers’ understanding of the need for change related to the Triple Aim, managers’ strategies, and staff and managers’ understanding of how the strategies would help achieve the Triple Aim. These were compared and discussed until consensus was established.

In the second analysis phase, we drew from the theory about mental models and approached the identification and development of second-order themes in a manner akin to root-cause analysis. We went through the first-order themes and repeated the question, “Why do they think like this?” To articulate the underlying mental models, we employed a graphical elicitation approach [20] and mapped potential second-order themes with a concept map, drawing to find patterns of how the themes related to each other. Inspired by modified analytic induction which has as its goal the identification of relationships between concepts through a process of development and testing of hypotheses derived from, for example, interview data [28, 29], we then combed through all the codes and meaning units to try and disprove each possible mental model. Through iterative cycles of articulation, mapping, and testing, discrepancies were identified, discussed, and resolved. The findings were discussed with the department managers (informant validation). Quotations were translated by a professional translator to improve flow without altering the meaning [30].

## Results

The staff group consisted of five males (all physicians) and thirteen females (four physicians, three nurses, three midwives, and three medical secretaries). In the manager group, all twelve interviewees were females (see Table 1). Ages were between 30–70.

**Table 1:** Interviewees’ profession, position and years of experience (*n* = 30)

Profession (Total number of staff)	Manager (years managerial experience)	Staff (years clinical experience)
Physicians (63)	1 Department manager (<5)	3 Obstetricians (>10)
		3 Gynecologists (1: 5–10, 2 > 10)
		3 Residents/interns (1 < 5, 2: 5–10)
Nurses (200)	1 Department manager (<5)	3 nurses (>10)
	4 Middle managers (3: 5–10, 1: >10)	
Midwives (130)	1 Department manager (5–10)	3 midwifes (1: 5–10, 2 > 10)
	4 Middle-managers (1: 5–10, 1:>10, 2: <5)	
Medical secretaries (30)	1 Middle manager (<5)	3 medical secretaries (>10)
Total: (423)	12	18

The findings are organized into two sections. In the first, we present the mental models that guide the different understanding of the Triple Aim. In the second, we illustrate how the mental models mediate the managers’ action strategies and staff response.

### Mental models associated with the triple Aim

Four mental models related to two themes: change drivers (C_1_ and C_2_) and economics (E_1_ and E_2_) were identified.

### Mental models of change

*Professional ethos* and *socio-political discourse* were the two mental models underlying how managers and staff understood what drives change.

#### Model C_1_: professional ethos

According to this mental model, *change in health care is driven by professionals’ desire to improve clinical outcomes and/or care and to get rid of old habits*. Change is the result of research, applications of evidence-based medicine, and advances in technology; made possible through a continuous process of professionally led critical reviews of clinical practice. In this model, there was no room for discussions about the economics of health care. Rounds of cost-saving initiatives from above were not seen as a way to advance healthcare:

[*Departmental activity*] is affected by new surgical techniques, which can enable patients to be discharged earlier, or [*new*] treatment protocols … I don’t think there is an overall strategy about this, there is no one who has thought, that if we do this or that, then… so I think it [change] will actually occur randomly. It is the progress or development that happens within these [clinical pathways] that really get to influence [the changes] and not a vision from above. (Gynecologist 2)

While all staff and managers shared this mental model, emphasis was on different dimensions of the Triple Aim depending on the profession. Nurses and midwives shared a focus on providing patients with security and comfort, e.g. “patients should be seen and heard as individuals”, whereas physicians were more focused on measurable clinical outcomes.

#### Model C_2_: socio-political discourse

According to this mental model, *change in health care is driven by the social and political discourse*. It is the external pressure linked to the societal discourse that has pushed health care to become more efficient and reduce costs. Politicians set the change agenda. Hospital management are then forced to finance political decisions through never-ending cost reductions:

[*The motive*] is cost savings from the regional council, yes, [*chuckle*] that’s the way it is… the State does not want to give enough much money to the new hospital. So, it’s all about cold hard cash, that’s it. (Medical Secretary 1)

Cost reductions were seen as a bitter necessity, but in a larger perspective, creating more value for money was perceived as fair:

We cannot allow ourselves to continue to be surprised that we need to save, because that's Denmark's agenda today. You have to realize you are a part of a greater cause. (Nurse middle manager 1)

Cost reduction was also perceived as misaligned with the core values of health care. The societal trend to increase efficiency was seen as potentially detrimental to the humanistic values of care. Both staff and managers felt that political decisions were often unrealistic, lacked contextual insight, and were mainly financially motivated.

When [*politicians*] look at the field, they look at the number of births. But they seldom look at the increase in the number of procedures for each birth, which require more and more staff. (Midwife middle manager 1)

### Mental models of economics

We identified two mental models underlying how managers and staff understood economics in health care that we labelled: “You-get-what-you-pay-for” or “More-bang-for-the-buck.”

#### Model E_1_: You-get-what-you-pay-for

According to this mental model, *there is a direct relationship between lower costs and lower quality*. This model was rampant among staff. This mental model can be described as that of an individual’s relationship to economics and can be likened to purchasing power at a grocery store – with less money one can only purchase goods or services of lower quality. Motivated by the intention to design and deliver top quality care, staff emphasized the need to prioritize and the potential negative consequences associated with cost savings.

Sometimes, I think that we are under too much pressure. It’s unrealistic to maintain quality under the new requirements because I think we already are efficient. (Obstetrician 3)

Staff was concerned about vulnerable and frail patients and that the increased focus on the most sick patients would compromise safety for “uncomplicated” patients. They felt that discussions about optimization encouraged a production focus that could conflict with a patient focus:

The vast majority are committed to the shortest possible stay, but… there are also a significant number of, in particular, cancer patients who express that while they feel pleased to be in a “national integrated cancer package”, they do not feel happy about being a “package”. (Gynecologist 3)

Staff worried about engagement in national organizations, research, and continuing education:

There is no time for research and development for the senior physicians, there is no possibility during working hours, so either they do it in their spare time or get financed by a third party. (Obstetrician 3)

Managers argued in a fashion similar to staff when they described for staff how they interacted with hospital management and politicians:

I think it is important that we open up about there being a limit to how ambitious we can be professionally in relation to the resources we have. This is something the politicians should know. (Department Manager 2)

Managers and staff described that politicians seemed reluctant to make the tough choices between patient groups, which could help guide managers and staff.

#### Model E_2_: more-bang-for-the-buck

According to this mental model, *cost constraints can contribute to improving care*.

Interviewees (mostly managers) described that they intuitively felt that something “good” had come from previous cost-saving rounds. In contrast to Model E_1_, this model was tied to a *hope* or a *belief* that benefits will continue to come. Managers saw opportunities to improve patient satisfaction and teamwork and bridge interdepartmental barriers:

…we're going to be a department that will be much more dynamic and team based… in the past we have had a very sharp division between gynecology and obstetrics and between sub-specialties. It will be softened by this change because we are simply so dependent on each other and of a cross-sectional dialogue. Helping each other, we are more likely to experience situations where we reach the limit of what we can accommodate, and then you have to look around to see where to get help. (Department manager 1)

Managers also expressed that while previous cost reductions had led to improved care additional cost-saving rounds would most likely follow. Thus, if the department was very effective at improvements the first time, there would be less leeway – a smaller “efficiency buffer” – to draw upon the next time, and then it would become harder to improve efficiency further.

Mental models mediate choice of and response to action strategies

A complex interplay could be discerned between the four mental models, which guided managers’ and staff actions as they faced the Triple Aim. They described the need for change as a result of external and political pressure to become more efficient juxtaposed with a professional desire to improve care. The models existed simultaneously in the organization and at times within the same individual. Staff and managers:

The motive for making changes in the department is a combination of our own desire to do things in an optimal way, and the constraint that “we do not have any more money than we have” – well there is this trend in society” (Gynecologist 3)

Managers voiced concerns that an externally imposed, top-down strategy would demotivate staff. They described how they translated the change imperative from a political pressure transmitted through the hospital’s top management (C2) into a focus on the improvement of clinical pathways (C1). A “patient first” perspective was described as a way to ensure high clinical quality and safety:

The focus is on the patient and not on a political decision and, in management, we are fully aware of where it [the decision] comes from, but we have not used it [the political pressure] very much in motivating employees to perform or create these new plans [for changes in patient pathways]. (Department manager 2)

They described their approach as taking the “professional path”:

What has been foremost in the change process is that we keep the focus on the professional. We take patient pathways as the point of departure, and review all the phases of the patients’ path in terms of if there is anything we can do differently. In contrast, we could have started from a [financial] frame. And, it's a very significant difference to choose such a change process. The result is that we focus much more on process and not so much on the result. It's been a conscious choice. (Department manager, 1)

Managers stressed the importance of paying attention to the staff concerns during the change process, not just optimization and efficiency. They explained that interdisciplinary involvement would motivate staff to change and positively affect the department as a consequence of staff working closely together and realizing the interdependencies between the wards, sections, and disciplines. Interdisciplinary workshops to generate improvement ideas were organized around patient processes, not departmental structures.

[Staff should] make suggestions on how we could do things differently, where we had some procedure that needed to be tightened up and we actually do some things just because we are used to doing it that way, there are many things that we really do just based on tradition and not because of any professional reason. (Department manager, 1)

The strategy was described as a way to placate both staff and hospital management – it enabled managers to respond to upper managerial pressures, but also to resonate with staff's intrinsic motivation. Moreover, in case of failure to achieve the cost savings, managers could argue that every stone in the department had been turned.

The interaction of the models did lead to frustration and anxiety. An opinion was that politicians only considered the number of patients and beds, rather than the complexity of the patient in the bed. The response for some staff was to replace their intrinsic motivation rooted in C_1_ with compliant behavior related to a feeling of being forced to change their ways of working:

We have merged [*sections*] over the past two years and have to be generalists rather than specialists… It's really hard to do things as well as before because now we have to be good at everything. (Nurse 3)

Models E_1_ and E_2_ drove understanding and strategies in different directions. The grocery store metaphor (E_1_) lead staff to doubt the effects of the planned changes and managers to say that they would accept lower service levels for patients. This led some staff to understand pursuit of the Triple Aim as aiming for the lowest acceptable level of quality, which negatively affected motivation. When managers described for staff their meetings with upper management, their statements also reflected E_1_. But E_2_ combined with C1 led managers to involve staff in mapping and reviewing clinical pathways instead of focusing on costs in order to generate energy, ideas, and commitment:

The biggest possibility is what we have now: the *esprit de corps* and staff inventing new things and thinking innovatively. It’s fun and it’s works and we have to continue with this, because then something new will pop up. (Department manager 3)

Thus, the understanding of the Triple Aim was influenced by which mental model was used by whom and under which circumstances: When the interaction between C_1_ and E_2_ dominated, the Triple Aim was understood as a way to improve care; in other combinations, the Triple Aim was perceived as a threat to clinical care and quality.

## Discussion

This study provides an empirical contribution to the scientific literature about how staff and managers understand the Triple Aim. On the surface, the understandings appears to follow the same professional demarcations which previous studies on logics, hybrid managers, and the relationship between economics and quality improvement in health care have identified [13, 15, 31, 32]. Physicians concentrated on the outcomes dimension and nurses on the experience dimension and both concentrated less on the cost aspects or the relationship between the dimensions. Staff considered the Triple Aim to be a dilemma, i.e. a question of either quality or cost savings, and managers saw it as a paradox, i.e. a possibility to improve and save costs. Underlying these divisions we identified four mental models of health care that appear to mediate staff and managers’ understanding of the change imperative inherent to the Triple Aim (Table 2).

**Table 2:** The mental models underlying staff and managers’ understanding of the Triple Aim

	Dominant among staff	Dominant among managers
Drivers of change in health care	C_1_	C_2_
	“Professional Ethos”	“Socio-political discourse”
	Change is due to new research, evidence & technology	Change is due to societal demands for efficiency
Economics of health care	E_1_	E_2_
	“You-get-what-you-pay-for”	“More-bang-for-the-buck”
	↓money → ↓quality	↓money → opportunity to ↑quality

**Fig. 1:**
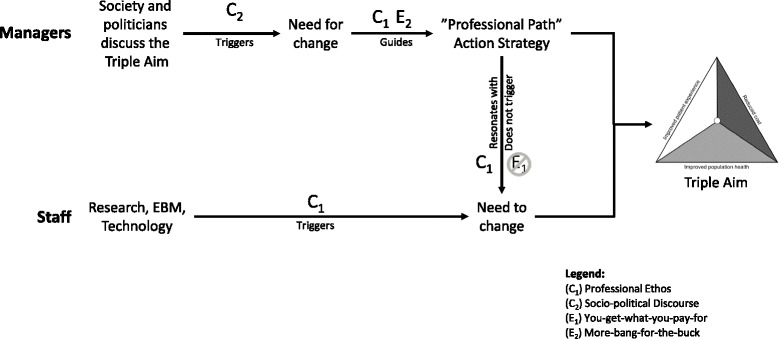
Simplified model of how mental models may mediate managers’ action strategies and staff response. Managers’ mental model of *Socio-political discourse* triggers awareness of the need for change. Mediated by managers’ mental models of *More-bang-for-the-buck* and *Professional ethos*, they choose a”Professional Path” action strategy that resonates with the staff *Professional Ethos* and does not trigger *You-Get-What-You-Pay-For*

Staff and managers understood the change imperative inherent to the Triple Aim mainly as a *political requirement* to become more efficient and reduce costs. This did not resonate with their mental model of what drives change in health care (C_1_). Managers, despite being triggered by the socio-political discourse (C_2_) and the mental model that economic pressure can lead to quality gains (E_2_), chose a strategy that resonated with (their own) professional ethos (C_1_). This could explain why a strategy similar to other QI and lean initiatives worked in this department. It appealed to diverse professional logics, but more importantly it resonated with the underlying mental model of change (C_1_), and mitigated the concerns triggered by E_1_ (Fig. 1).

The mental models on economics revealed a difficulty in relating quality with cost that mirrors the inconsistency of that relationship in the available research [7, 33]. Despite decades of QI applications in health care, none of the mental models captured the relationship as Deming described it. The simple and linear relationship between quality and cost from the perspective of a consumer at a grocery store (E_1_) is in direct contrast to Deming’s [5] view of quality improvement as a means to reduce costs. Model E_2_ suggests a reversed understanding of Deming´s “Chain reaction” model, as it begins with cost reduction instead of quality improvement.

IHI’s has called for new mental models to support achievement of the Triple Aim, such as “Compete on value, with continuous reduction in operating cost” and “Reorganize services to align with new payment systems” [16, 21]. While bringing attention to the need for new mental models, IHI actually describes strategies related to the socio-political discourse around management trends (i.e. value-based care) rather than mental models. In contrast, the empirically derived mental models of this study could help managers become more aware of the assumptions that underlie their choice of strategies.

Logics describe an “if-then” process of thinking often anchored in a profession. Differing logics may contribute to the management-medicine conflict, where executives adopt management trends to retain legitimacy [34]. Our findings suggest that attempts to marry logics, such as managerialism and medicine through hybridization, may be insufficient, because it is less a challenge of translation between logics and more a question of developing resonance with mental models of medicine. Too much focus on socio-political demands to optimize, application of a reverse-Deming logic, or talk about iterative cycles of measurement and improvement without relating to research, evidence-based medicine, and technological advances may prove to be another reason for failed QI initiatives [35, 36]. Without a deep understanding of C_1_ or E_1_, managers may inadvertently contribute to the development of compliant behavior and “resistance-to-change”. These behaviors can increase frustration, anxiety, and burnout [37].

Managers in pursuit of the Triple Aim should consider if the mental models identified in this study are applicable in their own setting. If they are, they should nuance their strategies so that they resonate with C_1_ and the different understandings of the cost-quality relationship. A “professional path” strategy could be a wise start. If the mental models are not applicable, managers should consider surfacing and articulating the mental models of their staff and develop strategies that resonate with these. Further studies could explore if an active and conscious contrasting of C_1_ with E_2_ could generate a “creative tension” that could lead to innovation [19].

### Methodological considerations

The main challenge to the trustworthiness of the findings is the risk of bias inherent to researching one’s own organization. To address this, the first author kept a journal throughout the study-period and continuously reflected on assumptions alone and with the co-authors to aid reflexivity. The journal made it possible to expose *a priori* understandings and assumptions, which could influence analyses while also making the author aware of deeper contextual understandings. The first author’s role was also continually reflected on in the research group and with one department manager. In addition, the four researchers individually reviewed and categorized the codes [38] into first-order themes before they were compared and discussed.

Specific contextual factors may have also influenced which mental models were surfaced during the interviews about the Triple Aim. The clearly defined stretch goals related to cost reduction and downsizing may partly explain the fact that two mental models were focused on economics. On the other hand, economics is a large part of both public and private health care systems. The managers were all clinically trained and most often active as medical professionals, which might explain their ability to draw from different mental models. In another context, where managers do not have a clinical background, this might not be the case.

The use of a single case allowed us to analyze in depth the mental models that mediated the choice of and response to action strategies. We randomized to select participants in order to capture a range of views from all professional categories and conducted many interviews to ensure saturation. We encourage others to repeat the study in other organizations striving towards the Triple Aim. Studies at other settings are needed to corroborate the effectiveness of the “professional path” strategy. As is the case with qualitative research, transferability is defined by how the results resonate with readers’ experience and understandings and if they apply the lessons learned in their own context.

## Conclusion

The interpretation of the Triple Aim as a paradox or a dilemma appears to be symptomatic of the conflict between four different mental models that guide staff and managers’ understanding of economics and change in health care. The mental models suggest divergent understandings of the quality-cost relationship and may provide a potential explanation of the practical challenges inherent to the Triple Aim. Change management practices may benefit from less focus on the symptoms of conflicting logics, but rather seek to articulate, test, and challenge our deeply held assumptions of how health care improves.

Managers should choose strategies that acknowledge and resonate with the mental models of staff and other managers. Pursuit of quality improvement in the face of financial constraints (the essence of the Triple Aim) requires attention to mental models. Managers might do well to translate the socio-political discourse, which often emphasizes money, into a change process that resonates with the mental models held by staff, who seem not to want to have money on their mind.
